# The Molecular Clock and Neurodegenerative Disease: A Stressful Time

**DOI:** 10.3389/fmolb.2021.644747

**Published:** 2021-03-26

**Authors:** Bethany Carter, Hannah S. Justin, Danielle Gulick, Joshua J. Gamsby

**Affiliations:** ^1^Gamsby Laboratory, USF Health Byrd Alzheimer’s Center and Research Institute, University of South Florida Health, Tampa, FL, United States; ^2^Department of Molecular Medicine, Morsani College of Medicine, University of South Florida, Tampa, FL, United States

**Keywords:** circadian, neurodegeneration, dementia, proteinopathy, stress

## Abstract

Circadian rhythm dysfunction occurs in both common and rare neurodegenerative diseases. This dysfunction manifests as sleep cycle mistiming, alterations in body temperature rhythms, and an increase in symptomatology during the early evening hours known as Sundown Syndrome. Disruption of circadian rhythm homeostasis has also been implicated in the etiology of neurodegenerative disease. Indeed, individuals exposed to a shifting schedule of sleep and activity, such as health care workers, are at a higher risk. Thus, a bidirectional relationship exists between the circadian system and neurodegeneration. At the heart of this crosstalk is the molecular circadian clock, which functions to regulate circadian rhythm homeostasis. Over the past decade, this connection has become a focal point of investigation as the molecular clock offers an attractive target to combat both neurodegenerative disease pathogenesis and circadian rhythm dysfunction, and a pivotal role for neuroinflammation and stress has been established. This review summarizes the contributions of molecular clock dysfunction to neurodegenerative disease etiology, as well as the mechanisms by which neurodegenerative diseases affect the molecular clock.

## Introduction

Circadian rhythms are daily cycles of biological processes in living organisms that are regulated endogenously and function to gate essential molecular, cellular, physiological, and behavioral activities to discrete times of the day. Circadian rhythms are defined by three properties: (1) They must have a period of approximately 24 h and continue to oscillate in the absence of external stimuli, (2) rhythmicity can be reset or “entrained” by external stimuli, and (3) they are temperature compensated or maintain periodicity regardless of temperature changes across the physiological permissible range (Dunlap et al., 2004). The central pacemaker in mammals, known as the suprachiasmatic nucleus (SCN), regulates the timing of circadian rhythms and resides in the anterior hypothalamus. The SCN controls output timing to peripheral oscillators, such as organ systems, to coordinate the daily cycling of numerous essential physiological processes. Examples of peripheral oscillators include the liver, heart and discrete regions of the brain, such as the hippocampus (Zhang and Sehgal, 2019). In healthy individuals this mechanism functions to regulate sleep–wake cycles, neuroendocrine activities, and memory (Moore, 2013; Sollars and Pickard, 2015).

As previously mentioned, an important feature of a circadian rhythm is that it can entrained by various external cues to allow an organism to adapt to changes in the environment. In humans, an example of this is light exposure when waking. Light, absorbed by intrinsically photosensitive retinal ganglion cells, signals through the retinohypothalamic tract to the SCN to reset the clock by adjusting the timing of the core molecular oscillator (reviewed below). The SCN then conveys this timing information to peripheral oscillators such as the heart, liver and lungs (Dibner et al., 2010; Richards and Gumz, 2012) via the immune, hormonal, and neurotransmitter signals to generate the proper timing of rhythmic physiological processes (Moore, 2013; Sollars and Pickard, 2015). When this system is perturbed, numerous adverse consequences can occur. For example, exposure to light at inappropriate times, such as prolonged light exposure to light-emitting electronic devices before bed, represses melatonin production and disrupts normal sleep patterns (Chang et al., 2015). Consistent perturbations of entrainment can lead to “circadian desynchrony,” a misalignment of the circadian clock with the external environment that is associated with an increased risk of numerous diseases and disorders such as cancer, diabetes, and Alzheimer’s disease (AD) (Sahar and Sassone-Corsi, 2009; Bass and Takahashi, 2010; Musiek and Holtzman, 2016; Logan and McClung, 2019). Additionally, extra-SCN clocks exist that can supersede or override the timing of the core clock (Anne-Marie Chang et al., 2014). For example, the hippocampus, which plays a central role in learning and memory function, has an entrainable circadian clock that can regulate the time-of-day changes in cognitive efficacy in the absence of descending signals from the SCN (Hasegawa et al., 2019).

### The Molecular Clock

**FIGURE 1:**
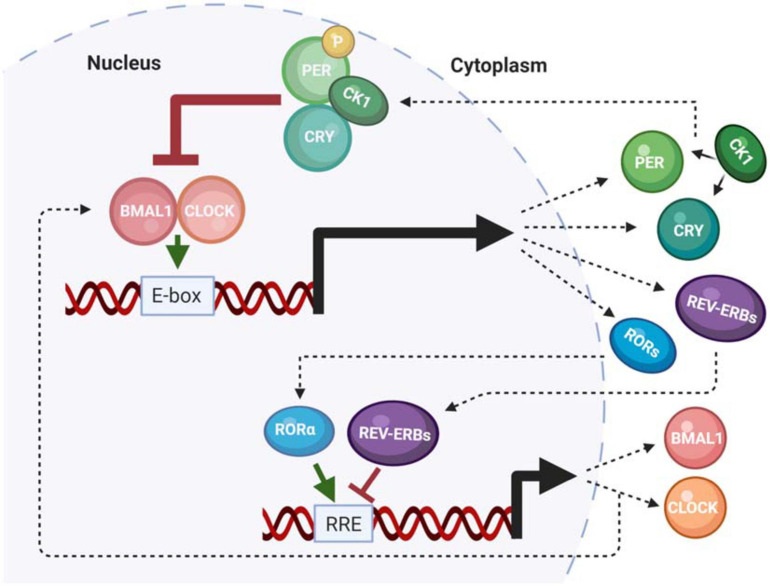
The mammalian molecular clock. A summary of the functionality of the mammalian core clock components that are impacted by neurodegenerative disease or play a role in pathogenesis.

Numerous excellent reviews have summarized our current understanding of the molecular circadian clock (Baker et al., 2012; Partch et al., 2014; Cohen and Golden, 2015; Takahashi, 2017; Patke et al., 2020). Therefore, this review will summarize the molecular components that are impacted by neurodegenerative disease. The molecular clock in humans is composed of a family of core clock genes that are involved in two interlocking feedback loops; a primary negative feedback loop, and a secondary stabilizing loop (Li et al., 2013; Patrick-Simon Welz, 2020). The primary is a transcription-translation feedback (TTFL) loop, which governs 24-h periodicity. Importantly, this aspect of the clock mechanism is conserved across almost all living organisms (Dunlap and Loros, 2017). The TTFL mechanism is initiated by the transcription factors Brain and Muscle Arnt-Like 1 (BMAL1) protein and Circadian Locomotor receptor Output Cycles Kaput (CLOCK), which heterodimerize then bind to enhancer box (e-box) consensus site sequences in the promoters of the *Period* (*Per1-3*) and *Cryptochrome* (*Cry1-2*) gene families. PER and CRY are then transcribed, translated, and ultimately shuttled back into the nucleus to bind to the heterodimeric BMAL1/CLOCK complex to repress their own transcription, as well as the transcription of any clock-controlled genes (CCGs) that also have e-box (or other clock-regulated) consensus sites. The secondary loop is centered on the production of BMAL1 and CLOCK. Here, rhythmic BMAL1 transcription is driven by the activation and repression of by the RAR-related orphan nuclear receptor (ROR) and Rev-Erb gene families, respectively, which competitively bind to retinoic acid response elements (RREs) within the BMAL1 promoter. Together, this system of feedback loops regulates periodicity, amplitude, and phase of the molecular clock (Figure 1) (Bae et al., 2001; Li et al., 2013). In addition to the roles of core clock genes, the molecular clock is regulated by rhythmic posttranslational modifications (PTMs). For example, isoforms of the casein kinase 1 (CK1) gene family phosphorylate PER in a time-of-day dependent manner to maintain proper clock timing (Etchegaray et al., 2009; Lee H. M. et al., 2011). An additional PTM that is characterized by the acetylation of CLOCK and BMAL1 mediates gene expression by interacting with histone acetyltransferases (HATs) (Masri and Sassone-Corsi, 2010; Zhang and Sehgal, 2019). All of these mechanisms function in concert to maintain the proper timing of the central and peripheral oscillators.

### Circadian Rhythms and Neurodegeneration

Neurodegenerative diseases are characterized by the progressive loss of neuronal structure and function, usually through neuronal death, and are caused by both genetic and environmental factors. The symptoms of neurodegeneration can include loss of motor control, cognitive decline, and mood changes, depending on the affected area of the nervous system. One of the early indicators that the circadian system is impacted by neurodegenerative disease was the observation that some patients have aggravated symptomology during the early evening hours, which is known as Sundown Syndrome. Sundowning is characterized by greater cognitive deficits and psychotic breakdowns during the evening hours, disturbed sleep patterns, and difficulty thermoregulating (Bliwise et al., 1995; Canevelli et al., 2016). These functions are all controlled by the SCN, indicating a connection between the cognitive and effective symptoms of dementia and the molecular clock.

Recent evidence has established a clear relationship between neurodegenerative phenotypes and the molecular clock (Chang and Guarente, 2013; Musiek et al., 2013; Musiek and Holtzman, 2016; Singh et al., 2018; Lananna and Musiek, 2020). Additionally, chronic perturbations in normal circadian rhythmicity are also believed to exacerbate neurodegenerative disease progression (Musiek, 2015). Thus, a bidirectional relationship between the clock and neurodegeneration exists. An example of this is the relationship between the clock and the adrenal glucocorticoid (GC) stress responses. The SCN regulates the hypothalamus–pituitary–adrenal (HPA) axis, driving the rhythmic release of adrenocorticotropic hormone (ACTH). This rhythmicity of ACTH has downstream effects on ACTH and GC receptor sensitivity that disinhibit the HPA axis during the waking phase; this disinhibition drives time-of-day dependent sensitivity to stress exposure. In return, stress increases the activation of the HPA axis, and the subsequent production of cortisol. This increased activation of the HPA axis has downstream effects on the clock by way of increased distal corticosteroids disrupting sleep (Han et al., 2012). If continual sleep disruptions persist, rhythms in the sleep–wake cycle itself will be altered, causing irregularities in core clock timing or circadian desynchrony (CD). Thus, the clock functions to regulate the stress response, and chronic stress can also affect the timing of the clock, which could be impacted by neurodegenerative disease pathology.

### The Clock, Output and Oxidative Stress

One of the major output pathways of the molecular clock feeds into cellular metabolism, including processes such as redox homeostasis and neuroinflammation. Disruption of redox homeostasis drives oxidative stress (OS), which is a key nexus between neurodegeneration and the circadian clock (Lananna and Musiek, 2020). BMAL1 drives the transcription of redox genes NAD(P)H quinone dehydrogenase and aldehyde dehydrogenase 2 (QR2) in the brain (Musiek et al., 2013), which play vital roles in preventing OS accumulation and neuronal damage (Musiek et al., 2013; Musiek and Holtzman, 2016). TTFL factors also mediate antioxidant activity through their interactions with NAD-dependent deacetylase sirtuin 1 (SIRT1) (Asher et al., 2008) which is heavily involved in maintaining cellular redox homeostasis (Asher et al., 2008). SIRT1 activates the transcription of both BMAL1 and CLOCK in the SCN (Chang and Guarente, 2013). Its dysfunction is a hallmark of Parkinson’s disease (PD) (Singh et al., 2017), and emerging evidence also points to its role in AD pathogenesis. Together, these findings present a mechanism to explain the circadian impact on the onset and development of multiple neurodegenerative diseases and suggest a significant bidirectional relationship between neurodegeneration and circadian desynchrony that may provide a target to reduce the risk or severity of neurodegenerative diseases.

In this review, we will address the role of the circadian clock in the development and progression of both common and rare neurodegenerative diseases - specifically in AD, Lewy body dementia (LBD), PD, Huntington’s disease (HD), progressive supranuclear palsy (PSP), Pick’s disease (PiD), and frontotemporal lobar degeneration-tau (FTLD-tau). As the neuropathology of each disease covered is central to the circadian rhythm disorders observed in patients, we have chosen to group each neurodegenerative disease by their associated proteinopathies.

## α-Synuclein Proteinopathies

Lewy body dementia and PD are both characterized by neuronal α-synuclein inclusions known as Lewy bodies. The normal function of α-synuclein is not well-understood, but evidence suggests a role in synaptic vesicle mobility (Scott and Roy, 2012). Misfolded α-synuclein results in β-sheet aggregates, which are hypothesized to spread in a prion-like manner causing devastating neuronal damage (Ma et al., 2019). In prion diseases, misfolded proteins cause the misfolding of their normal protein counterpart (Walker et al., 2015).

### Parkinson’s Disease

Parkinson’s disease is a neurological motor disorder characterized by uncontrollable tremors and difficulty initiating voluntary movements. Presently, the precise etiology of PD is unclear, although genetics can play a central a role – specifically through the mutation of the *Parkin* gene (*PRKN*). During PD pathogenesis, significant cell death occurs among dopamine producing neurons of the substantia nigra, leading to this loss of motor function. Lewy bodies aggregate in the basal ganglia and neocortex (Walker et al., 2015). As it progresses, patients with PD develop cognitive decline, dementia, as well as mood and behavioral disorders. Additionally, circadian dysfunction also occurs in PD and is characterized by many sleep-related pathologies, such as excessive drowsiness, insomnia, night terrors, REM cycle disruptions, and sleep fragmentation (Petit et al., 2017; Trenkwalder et al., 2017). Furthermore, circadian dysfunction coupled with neuroinflammation is a risk factor for PD. Lauretti et al. (2017) subjected C57BL/6 mice to abnormal light-dark cycles (20:4 LD) prior to administering a PD-inducing neurotoxin. Circadian-disrupted mice had severe neuroinflammation and a greater degradation of motor skills, indicating that circadian dysfunction is a potential risk factor for PD. This effect was recapitulated in a rat model, where PD pathology was induced or exacerbated in one of three ways: by injection of lipopolysaccharide, injection of rotenone, or injection of both reagents (Li et al., 2019). All three groups exhibited similar loss of dopaminergic neurons and neuroinflammation. Of the nine core clock genes evaluated, six had decreased expression levels in all three groups. Circadian disturbances have also been shown in an α-synuclein overexpressing (ASO) mouse model of PD (Kudo et al., 2011). There were selective deficits in circadian activity such as lower waking activities and more fragmented sleep patterns. ASO mice had normal *Per2* expression and lower daytime firing rates in the SCN neurons, which have also been observed in HD models (Smarr et al., 2019). Importantly, ASO mice showed no negative effects in their light adaptation responses, indicating that photic entrainment remained intact.

There is evidence that PD and the molecular clock are also connected at the genetic level (Gu et al., 2015). A screen of eight key core clock genes in sizable populations of PD patients and healthy controls revealed a significant association between PD risk and clock gene SNPs. Of all the screened genes, *BMAL1* and *Per1* had the greatest number of PD-associated SNPs. Different PD subtypes were also correlated with specific haplotypes in the identified *BMAL1* SNPs. This indicates that SNPs in TTFL genes are risk factors for PD that could potentially serve as biomarkers to aid PD diagnosis and treatment. However, the core clock may be affected by the same factors that increase the risk of developing PD. For example, the *PRKN* mutation may influence crosstalk between the TTFL and mitochondrial bioenergetic pathways. Fibroblasts taken from two genetic PD patients exhibited dampened oscillations in bioenergetic activities, reduced rates of mitochondrial oxygen consumption, and dysregulated TTFL factor expression patterns - CLOCK, CRY1, and CRY2 were upregulated while PER2 was downregulated in these cells (Pacelli et al., 2019). Epigenetic modifications may also play a role in aberrant clock gene expressions. A screen of seven key TTFL genes in the leukocytes of PD patients showed that the *Neuronal PAS Domain Protein 2* (*NPAS2*) promoter is hypomethylated compared to healthy controls. NPAS2 performs a similar function to CLOCK, as it can heterodimerize with BMAL1 to drive circadian rhythmicity. *Cry1* conversely showed greater promoter methylation, while none of the remaining five genes showed any change in methylation (Lin et al., 2012).

In addition to increasing clinical evidence that human PD patients exhibit deregulation of BMAL1 (Breen et al., 2014; Gu et al., 2015), there is a decrease in expression of BMAL2 which is a paralog of BMAL1 (Ding et al., 2011). BMAL2 is not essential for normal clock functionality, but knockout of BMAL1 can be rescued by the constitutive expression of BMAL2 in mice, indicating paralog compensation. Additionally, Gu et al. (2015) speculate that lowered BMAL1 levels are a result of PD-induced dopamine deficiency. This postulation is supported by studies in a rat model of PD (Gu et al., 2015). Here, 6-hydroxydopamine (6-OHDA), a potent neurotoxin that destroys dopaminergic and noradrenergic neurons, induced a PD-like phenotype, which caused an increase in levels of acetylated SIRT1 and BMAL1, decreased *Per* and *Cry* expression, and an alteration of neuronal antioxidant activity (Wang et al., 2018). Furthermore, treatment with the antioxidant Resveratrol partially rescued this abnormal phenotype. Based on these results, it is possible that core clock abnormalities contribute to reduced antioxidant capacity in PD. To further outline the potential circadian component in PD, a study from Liu et al. (2020) shows inactivation of BMAL 1 in MPTP-treated mice results in significant reduction of dopaminergic neurons, and decreased levels of tyrosine hydroxylase, dopamine, and 3,4-dihydroxyhenylacetic acid content in experimental mice. Increased activation of the neuroinflammatory response was observed *in vivo* and *in vitro*, thereby suggesting BMAL1 may have a neuroprotective effect in the depletion of dopaminergic neurons and disrupted BMAL1 may accelerate PD pathology (Liu et al., 2020). “These studies taken as a whole suggest that selectively targeting TTFL factors with pharmaceuticals or other chronotherapies may succeed in alleviating symptoms in PD patients. However, inconsistencies concerning the impact of PD on the core clock components clouds our current understanding of the relationship between PD and the clock. Further investigation of this connection is needed before a therapeutic strategy can be developed.”

### Lewy Body Dementia

LBDs are the second most common type of degenerative dementia affecting the elderly (Petit et al., 2017). A pilot study investigating clock gene disturbances in dementia patients found that LBD patients had a higher frequency of aberrant methylation patterns of core clock genes compared to other dementia patients (Liu et al., 2008). Among the nine genes examined, *Per1* and *Cry1* were methylated in dementia patients but not in healthy controls. LBD patients had somewhat greater disturbances of locomotor circadian rhythmicity than AD patients (AD is discussed in further detail below); however, there was no association between behavioral disturbances and gene methylation in any of the dementia groups (Liu et al., 2008). Interestingly, the circadian sleep disturbances associated with LBD appear more severe than in AD, which was initially shown by Harper et al. (2004). This data was supported by more recent findings where a 70% increase in REM sleep behavior disorder (RBD)-like symptoms, including increased daytime drowsiness, was observed in LBD as compared to AD and controls (Cagnin et al., 2017). However, further research is necessary to understand the molecular mechanisms that underlie these behavioral observations. RBD has been proposed to be a precursor stage of synucleinopathies, some patients exhibiting signs of RBD full years prior to any neurodegenerative symptoms (Brown, 1994; Zhang et al., 2020). To evaluate the involvement of clock genes in RBD, Weissova et al. (2018) investigated 24-h melatonin blood profiles in RBD patients and healthy individuals. RBD patients did not have circadian rhythmicity for key clock genes Per2, BMAL1, and rev-Erbα, as compared to healthy individuals. Additionally, melatonin profiles of RBD patients were delayed by 2 h, and habitual sleep phases were delayed by 1 h. These results suggest that altered expression of *Per2*, *BMAL1*, and *Rev-Erb*α combined with delayed melatonin secretion could be responsible for RBD (Weissova et al., 2018). Interestingly, melatonin may also be involved in the progression of HD, discussed later in this review.

## Tauopathies and β-Amyloidosis

Tauopathies are a class of neurodegenerative diseases that arise when the protein tau, which functions to stabilize the microtubules of neurons, becomes misfolded and forms pathogenic intracellular aggregates that cause neurodegeneration (Irwin, 2016) and contribute to memory cognitive deficits (Lee V. M. et al., 2011). Genetic abnormalities such as missense mutations, SNPs, pathogenic haplotypes, and deregulated alternative splicing have all been implicated in tau pathogenesis (Caillet-Boudin et al., 2015; Irwin, 2016). These changes can induce structural instability, reduce tau’s microtubule binding capacity (Strang et al., 2019), increase intracellular concentrations of free-floating phosphorylated Tau (pTau), and contribute to the formation of neurofibrillary tangles (NFTs). Clinical evidence indicates that the SCN is also damaged by tau aggregation (Stopa et al., 1999) and circadian disturbances in the behavior of tauopathy patients suggest an imbalance in the TTFL (Anderson et al., 2009). In addition to tauopathy, a defining hallmark of some neurodegenerative diseases, like AD, is the presence of β-amyloid plaques, which are composed of toxic extracellular aggregates of amyloid beta (Aβ). The amyloid precursor protein (APP) is cleaved by both β-secretase (BACE1) and γ-secretase to generate Aβ and are associated with the secretion and aggregation of toxic Aβ (Luo et al., 2001; Steiner et al., 2018; Yuksel and Tacal, 2019). As amyloid plaques aggregate, they can also exacerbate tauopathy, driving further pathogenesis (Gan et al., 2019; Busche and Hyman, 2020).

### Alzheimer’s Disease

Alzheimer’s disease is one of the most heavily researched neurodegenerative diseases due to its rapidly increasing prevalence, mostly because humans are living longer and AD is primarily a disease that affects those 65 years of age and older (Xia et al., 2018). In the United States alone, AD cases are projected to reach 14 million by 2050 (Hebert et al., 2013). Risk factors such as aging and OS are both tightly intertwined with the core clock. It is well known that aging leads to oxidative damage (Zhang et al., 2015; Gomes et al., 2017; Mecocci et al., 2018). In normal aging, several cellular processes are attenuated over time. This senescence is caused by a chronic state of low-level stress, induced by both genetic and environmental factors. However, AD patients generally have markedly higher OS levels, which are thought to contribute to more rapid degeneration (Chen and Zhong, 2014). In healthy individuals, TTFL factors mediate antioxidant processes in many organ systems, and the maintenance of redox homeostasis in the brain is essential in staving off neurodegeneration. It is unclear whether OS causally contributes to circadian dysfunction or vice versa. Most likely, feedback between the two pathologies contributes to both. Thus far, changes in the expression of PER and BMAL1 seem to have the strongest connections to aging and OS.

As previously mentioned, the TTFL is an evolutionarily conserved mechanism. Therefore, much insight into the relationship between the clock and neurodegenerative disease can be gained by evaluating this relationship in an organism with much simpler genetics and nervous system. For example, in the model organism *Drosophila melanogaster* there is only one *Per* gene, whereas in mammals there are three (*Per1–3*). This redundancy can lead to confounds such as the paralog compensation (Baggs et al., 2009). In *Drosophila, Per* regulates sleep patterns and working memory (Dissel et al., 2015). Furthermore, high expression of PER is associated with neuroprotective effects against oxidative damage (Krishnan et al., 2009). Ablation of *Per* accelerates neurodegeneration (Krishnan et al., 2009, 2012). When subjected to hyperoxia, *Per* knockout flies had greater oxidative damage, impaired antioxidant stress responses, neurodegeneration, and shortened lifespans. OS also induces aberrant sleep patterns such as those seen in aged AD patients. Over time, the sleep–wake cycle amplitudes decreased in OS-challenged flies, and the circadian rhythm loses its endogenous control, becoming temperature-dependent (Koh et al., 2006). Flies in a cooler environment also showed markedly slower neuronal degeneration during sleep cycles. This could be related to the lowering of core body temperature that occurs in healthy mammalian sleep cycles (Harding et al., 2019). Additionally, age-related losses of circadian rhythmicity in peripheral tissues have been recapitulated in other *Drosophila* studies (Chen et al., 2014; Long et al., 2014; Kuintzle et al., 2017). One such study by Buhl et al. (2019) demonstrates that the 0N4R isoform of Tau causes behavioral changes which match those of human AD patients. Additionally, a hyperactive circadian phenotype was observed, and then validated by whole-cell current clamp recordings of large lateral ventral neurons. These findings showed an especially depolarized membrane resting potential, and higher spontaneous firing rate among transgenic flies. Interestingly, input resistance, which is expected to cycle in wild type flies, maintained the same magnitude during the day and nighttime in the experimental group. These findings further outline the relationship between Tau isoforms and circadian disruption (Buhl et al., 2019).

Amyloid beta can also alter PER1 and PER2 expression in both the central oscillator (SCN) and peripheral cardiovascular tissues (Wang et al., 2016). Per is essential for maintaining healthy cardiac function and alterations in PER expression are strongly correlated with cardiac dysfunction (Young, 2006; Maemura et al., 2007). Similar patterns of PER expression may also exist in AD patients, as cardiovascular co-morbidities, such as atherosclerosis, are thought to be risk factors for AD because of the damage inflicted on the cerebrovascular system (Iadecola, 2003; Roher et al., 2011). Another potential player in these interactions is SIRT1 (Bellanti et al., 2017). A mouse model of AD that has both Aβ and tauopathy phenotypes (3xTG-AD) display altered hippocampal expression of SIRT1 after different patterns of light exposure (Bellanti et al., 2017) indicating circadian desynchrony influences OS pathways. SIRT1 even mediates transcriptional control of TTFL genes and this process is attenuated by aging (Chang and Guarente, 2013). These findings underscore the importance of OS pathways in circadian dysfunction (Song et al., 2015).

Evidence suggests that loss of rhythmicity contributes to Aβ production [by BACE1 (Ma et al., 2016)] and accumulation, and the reverse is also true - Aβ alters BMAL1 degradation (Song et al., 2015), deregulating the molecular clock. Exemplifying the bidirectional relationship between Aβ and BMAL, Kress et al. (2018), demonstrated that complete BMAL Knockout produces a rhythmic behavior in the deposition of AB plaque, and that local BMAL Knockout increases AB plaque burden. In addition, the circadian CK1 enzymes modulate Aβ activity by increasing the production of Aβ (Oumata et al., 2008; Ooms and Ju, 2016). BMAL1 levels are affected by Aβ as shown by Song et al. (2015) where *in vitro* Aβ induces degradation of BMAL1 and CBP via PTM SUMOylation. Consequently, PER2 expression was deregulated, corroborating the disrupted expression of BMAL1 and PER2 that was observed *in vivo*. Furthermore, knockdown of Sumo1 rescued the degradation of BMAL1. Given that Aβ over-accumulation increases ROS production (Smith et al., 2007) and that neuronal redox homeostasis is regulated by BMAL1/CLOCK (Musiek et al., 2013), the molecular dysfunction of the TTFL may contribute to Aβ-mediated neuronal cell death. On the other hand, circadian desynchrony may not be a direct product of pathogenic Aβ, but of some underlying mechanism that also increases Aβ production. Flies that overexpress the *Drosophila* homolog for APP in their central pacemaker neurons maintain robust circadian rhythms in spite of aging (Blake et al., 2015). However, overexpression of *Drosophila* β-secretase led to disturbed rest/activity rhythms, decreased PER expression and dampened oscillations of PER in the central pacemaker neurons. These effects were most prominent in aging flies, suggesting that β-secretase mediated circadian dysfunction is exacerbated in an age-dependent manner.

BMAL1 is neuroprotective against aging-induced neuronal decline, and yet BMAL1 is itself affected by aging and oxidative damage. BMAL1 knockout mice display an early aging phenotype characterized by sarcopenia, decreased hair regrowth, and increased ROS levels in the heart, kidneys, and spleen (Kondratova et al., 2010). BMAL1 deficiency also impairs PER and CRY expression, habituation, neuronal ROS homeostasis, and causes hyperactivity (Kondratova et al., 2010). Additionally, BMAL1 knockout mice also exhibit severe reactive astrogliosis in cortical and hippocampal tissue, while single knockouts of clock genes *Per1*, *Per2*, *CLOCK*, or *Npas2* produced no astrogliosis (Musiek et al., 2013; Musiek and Holtzman, 2016). Astrogliosis is an increase in the number of astrocytes, which are important cellular mediators of the neuroimmune response. Both global and neuronal deletion of BMAL1 produced astrogliosis, indicating a role for neuronal BMAL1 in this effect on astrocytes. Interestingly, SCN astrocytes have a significant impact upon neuronal activity during the circadian rest phase by inhibiting neuronal activity (Brancaccio et al., 2017). SCN astrocyte rhythmicity is also suspected to determine period length of locomotor activity. In an experiment conducted by Tso et al. (2017), it was found that knocking out astrocyte-specific BMAL1 lengthened the period of wheel running activity. Furthermore, combined knockout of CLOCK and Npas2 produced similar astrocyte activation to the BMAL1 KO mice, suggesting that dysfunction in the positive arm of the TTFL plays a vital role in the development of this age-dependent neuropathology. An aged hamster model also demonstrated attenuated BMAL1 expression across many regions of the brain, particularly in extra-SCN tissues (Duncan et al., 2013). Researchers postulated that this subsequent weakening of clock gene expression in regions of the brain might contribute to age-related cognitive deficits. Taken together, these findings suggest that circadian dysfunction has a clear impact on memory deficits and that natural aging processes are associated with decreased BMAL1 expression. In addition to BMAL1’s role in astrogliosis, a recent report has revealed further evidence for the relationship between the clock and astrocyte function. Lananna et al. (2020) show that the clock regulates the production of *Chi3l1*, which encodes for YKL-40, a glycoprotein biomarker for neuroinflammation in CSF that is increased in AD patients. The authors observed that *Chi3l1* is not only regulated by the core clock, but that deletion of Chi3l1/YKL-40 reduced amyloid plaque formation and Aβ phagocytosis (Lananna et al., 2020). This exciting result suggests that deregulation of a CCG such as *Chi2l1* might lead to AD pathogenesis.

In addition to role of core clock genes involved in transcriptional activation and transcriptional repression, core clock genes involved in post translational modifications are also impacted by AD. CK1δ may contribute to the aggregation of hyperphosphorylated tau inclusions (Li et al., 2004) and associates with NFTs in several neurodegenerative diseases, such as AD (Ghoshal et al., 1999; Schwab et al., 2000) PD, PSP, and PiD (Schwab et al., 2000). Among the dementia patients studied by Schwab et al. (2000), CK1δ did not associate with tau-negative pathogenic inclusions (e.g., Lewy bodies, Marinesco bodies) of non-tauopathy neurodegenerative diseases, whereas it had variable immunostaining intensity amongst dystrophic neurites, NFTs, and neuropil threads in AD brains (Schwab et al., 2000). Thus, it is possible that CK1δ accumulation could serve as a clinical biomarker for tau-specific neurodegenerative lesions. CK1δ isozymes may also directly influence protein turnover in the autophagic and phagocytotic processes that are essential in maintaining neuronal health. The dysfunction of these processes is widely implicated in neurodegeneration (Menzies et al., 2015, 2017; Martini-Stoica et al., 2016; Mecocci et al., 2018). Future research is needed to clarify the mechanisms of CK1δ-related accumulation in pathogenic tau plaques, although some work suggests a possible interaction with transactive response DNA-binding protein (TDP-43), another hallmark of neurodegeneration. CK1δ phosphorylates TDP-43 and promote its mislocalization (Nonaka et al., 2014; Gu et al., 2020), aggregating with TDP-43 and tau in granulovacuolar degeneration bodies (GVBs) of the cytoplasm, to promote tau hyperphosphorylation (Ghoshal et al., 1999; Yamazaki et al., 2011). Of note, CK1δ also contributes to neurodegenerative diseases through amyloidosis. We have previously studied this role in APP-PS1 mice, which manifest an abnormal sleep–wake rhythm and impaired cognition for tasks that rely on the prefrontal cortex and hippocampus (Sundaram et al., 2019). Treatment of this model with a selective CK1 δ/ε inhibitor resulted in a dose-dependent reduction of Aβ burden in the hippocampus and prefrontal cortex compared to vehicle-treated littermates. Additionally, the CK1 inhibitor reduced Aβ plaque size in the prefrontal cortex and hippocampus, increased period length and rescued some cognitive function. Since CK1 indirectly controls Aβ formation by phosphorylating APP and its subsequent proteases β and γ secretase (Chen et al., 2017), targeting its activity with pharmaceuticals may be a promising avenue of treatment for tauopathies that co-occur with amyloidosis. The *Drosophila* ortholog of CK1δ, Doubletime (Dblt), can shorten period length when mutated. Findings from *Drosophila* suggest a further interaction between CK1δ and tau. Means et al. (2015) knocked down Spaghetti, an HSP-90 co-chaperone that upregulates Dblt, and observed both defects in dClk expression, and upregulation of the initiator caspase Dronc that cleaves tau and accelerates neurodegeneration.

Less studied, but essential to this discussion is the influence of aberrant epigenetic modifications on TTFL factors. For example, oscillatory DNA methylation contributes to the regulation of BMAL1 (Cronin et al., 2017). Researchers increased methylation in NIH3T3 fibroblasts using *S*-adenosyl-methionine, a methyl donor. This led to decreased amplitude, longer periods, and phase delays in circadian rhythmicity, while treatment with a DNA methyltransferase inhibitor produced the exact opposite effects (Cronin et al., 2017). A subsequent postmortem analysis of midfrontal cortical tissue from deceased AD patients revealed abnormal oscillatory methylation of the BMAL1 promoter compared to healthy controls (Cronin et al., 2017).

### Frontotemporal Lobar Degeneration-Tau

Frontotemporal lobar degeneration-tau is a less common dementia that is nonetheless characterized by hyperphosphorylated tau deposits, often secondary to tau mutations (Bodea et al., 2016). This is subcategory of frontotemporal dementia (FTD), which is a broad term to designate neurodegeneration that produces pathology in discrete regions of the brain as well as distinct behavioral, emotional, language, and motor impairments (Bang et al., 2015). Although FTDs encompass a wide range of neurodegenerative diseases, there is clear clinical evidence of associated circadian dysfunction across this spectrum. FTD patients have comparable circadian symptoms to AD patients, such as increased difficulty falling and staying asleep, persistent daytime fatigue (Sani et al., 2019), and declines in sleep continuity (Kundermann et al., 2011). Additionally, our own research demonstrates impaired circadian functionality in a transgenic tau mouse model of FTLD-tau, which expresses a human mutant tau allele and is known as the Tg4510 line (Stevanovic et al., 2017). Tg4510 mice display a long free-running period in activity patterns as well as alterations in the circadian timing of BMAL1 expression in the hippocampus, and PER2 expression in the hippocampus and hypothalamus (Stevanovic et al., 2017). These defects may be due to the presence of elevated pTau in the SCN, which was also noted in this study. Furthermore, recent evidence demonstrates impaired cytoplasmic homeostasis, caused by tauopathy (i.e., elevated pTau), might impede PER trafficking into the nucleus of neurons of the SCN, ultimately leading to circadian rhythm dysfunction (Beesley et al., 2017). Clinical evidence also indicates that the SCN is also damaged by tau aggregation (Stopa et al., 1999) and circadian disturbances in the behavior of tauopathy patients suggest an imbalance in the TTFL (Anderson et al., 2009). Additionally, human tissue studies have proposed a link between dipeptide repeat (DPR) protein inclusions and sleep–wake disturbances in FTLD patients, as well as severe sleep disturbances and possible phase delay indicated by sleep diary data in human FTD cohorts (Anderson et al., 2009; Dedeene et al., 2019). However, a clear understanding as to how the clock is impacted by FTD is obscured by the paucity of information on this topic.

### Pick’s Disease and Progressive Supranuclear Palsy

Similar to other tauopathies, CK1δ was found in NFTs in PSP and associated with Pick bodies in PiD (Schwab et al., 2000). PSP patients share similar clinical phenotypes with PD patients, including poor sleep quality, obstructive sleep apnea, restless leg syndrome and difficulty falling asleep and staying asleep (Gama et al., 2010; Walsh et al., 2017). These conditions may exacerbate underlying circadian dysfunction, as restless leg syndrome was associated with sleep deficits in PSP. The circadian activity rhythms in PSP patients also have decreased amplitude and inter-daily stability compared to healthy controls (Walsh et al., 2016). Additionally, there is a positive correlation between PSP severity and lower relative and absolute amplitudes of circadian activity rhythms. However, there was no association between intra-daily rhythm variability or inter-daily rhythm stability and disease severity (Walsh et al., 2016). Therefore, it is unclear whether or not circadian dysfunction influences PSP severity.

## Huntington’s Disease

Huntington’s disease is a rare genetic neurodegenerative disease in which the Huntingtin (HTT) has an abnormal expansion of 40 or more CAG trinucleotide repeats (Orr and Zoghbi, 2007). HD causes severe cognitive decline into dementia, locomotor impairments, circadian dysfunction, and psychotic episodes (Morton, 2013). Onset occurs during middle age and is generally fatal within 10 years of diagnosis. Like other neurodegenerative diseases, HD has no cure and shares similar sleep and circadian rhythm disturbances, which associate with greater cognitive impairment and depression (Aziz et al., 2010).

In *Drosophila*, TTFL proteins can affect the aggregation of mutant Huntingtin (mHTT) protein. A partial knockdown of *Drosophila* CLOCK (dClk), an ortholog of the mammalian *Clock* gene, is suppressed by mHTT aggregation. However, this neuroprotective effect was lost in the absence of *Per*, which was drastically reduced during its normal peak hours (Xu et al., 2019). Additionally, preclinical HD models show improvements with chronotherapy. For example, light therapy improves some forms of circadian desynchrony in several different HD mouse models (Wood et al., 2013; Ouk et al., 2017; Wang et al., 2017). After receiving 6 h of daily blue light exposure, two mouse models of HD, Q175 and BACHD, had improved locomotor activity, although sleep patterns were unaffected (Wang et al., 2017). The R6/2 mouse also has a positive response to light therapy. HD-induced circadian dysfunction was considerably delayed phase shifts (Wood et al., 2013). In one case, a prolonged photoperiod (16:8 LD) extended lifespan and improved nocturnal behavior rhythms while the opposite pattern (8:16 LD) decreased survival (Ouk et al., 2017). Interestingly, the mice were still able to adapt to 4-h phase advances and delays (Wood et al., 2013), indicating increased reliance on external cues during entrainment. However, a *Drosophila* model of HD demonstrated conflicting evidence. Mutant flies had abnormally prolonged expression of PER and TIMELESS (another component of the *Drosophila* negative arm) and hallmark circadian activity disturbances (Farago et al., 2019) indicating a disrupted TTFL In mammals, PER1 and PER2 are also important players in photic entrainment., and both are deregulated in symptomatic HD mice with retinal degeneration (Morton et al., 2005). As a result, mice have reduced light sensitivity. Furthermore, benzodiazepine treatment rescues abnormal PER2 expression in R2/6 mice, thus improving circadian function (Pallier et al., 2007).

The rhythmicity of peripheral oscillators is also affected by HD. Q175 mice have blunted cardiac and body temperature rhythms, impaired autonomic nervous system function, and a completely ablated sympathetic nervous system (Smarr et al., 2019). A newly emerging chronotherapy is time restricted feeding (TRF), in which eating is restricted to a 6- to 8-h time window (Manoogian and Panda, 2017). This method has seen some success in Q175 mice in the form of improved locomotor rhythms and restored autonomic nervous system functionality (Wang et al., 2018). TRF also restores peripheral clock gene expression and ameliorates metabolic dysfunction in the livers of R2/6 mice (Maywood et al., 2010). This HD model is characterized by irregular *Cry* and *Per* rhythms and the preliminary success of TRF indicates that these genes are positively affected by treatment.

Recently, melatonin has been found to be produced in neuronal mitochondria (Suofu et al., 2017). Melatonin is well known to be circadian regulated, and signals darkness to the SCN. In a study conducted by Jauhari et al. (2020), it is demonstrated that HD (AANAT knockout) mice exhibit increased levels of mtDNA release, cGAS activation, and inflammation, all of which can be modulated by exogenous melatonin. Melatonin deficient mice exhibited accelerated aging and neurodegenerative pathology. This mtDNA activation of neuronal proinflammatory response provides potential insight to the circadian involvement in HD (Suofu et al., 2017; Jauhari et al., 2020). Although the precise molecular relationship between CD and HD remains unclear, the association between the onset of HD and dysfunction of the molecular clock is not. CD likely contributes to the pathology of HD by way of chronic inflammation, mitochondrial dysfunction, and DNA damage. This suggests a codependent relationship between HD and CD, where HD onset may present with symptoms of CD, and CD can accelerate the development of HD (Kuljis et al., 2012).

## Proteinopathies, Neuroinflammation, and the Clock

As previously described, one important function of the clock is to mediate the timing of specific activities of the immune system. These essential activities include leukocyte recruitment, cytokine production, and cell proliferation (Scheiermann et al., 2013). The reverse is also true. In an excellent review, Segal et al. (2018) demonstrate that immune mediators can also alter clock gene expression. Several excellent sources have outlined the circadian influence over multiple cytokines, chemokines, and hormones linked to the pro-inflammatory response. CLOCK and CRY both activate protein complexes essential to the production of cytokines and chemokines, and BMAL1 can both inhibit CLOCK’s activation of these complexes and bind to e-boxes in promoter regions of immune mediator genes to produce rhythmic levels of cytokines and chemokines (Segal et al., 2018). These changes produce higher levels of cytokines and chemokines important to inflammatory responses during the active phase (Segal et al., 2018).

Neuroinflammation is integral to the onset and progression of several neurodegenerative diseases. In the healthy brain, neuroinflammation is kept in check by anti-inflammatory signals. This process is essential because there is little neurogenesis to replace damaged neurons in the adult brain. Thus, aging and disease-related processes that shift the balance toward pro-inflammatory signaling will increase neuron loss. A strong association between the neuroinflammatory response and the core clock has been established through Rev-Erbα. Recent studies have shown that Rev-Erbα is essential to microglial activation and neuroinflammation and is directly mediated by BMAL1. Microglia are the specialized immune cells of the central nervous system; in a healthy brain, they are primarily anti-inflammatory. Furthermore, microglia have a functional molecular clock and express PER1, PER2, Rev-Erbα, and BMAL1 in a rhythmic manner that is disrupted by aging (Fonken et al., 2016). In a study conducted by Griffin et al. (2019), Rev-Erbα had a strong connection to neuroinflammation by a series of *in vivo* and *in vitro* experiments. *Rev-Erb*α deletion induced spontaneous hippocampal microgliosis (microglia activation). *Rev-Erb*α deletion also exacerbated LPS-induced neuroinflammation. However, when injected twice daily with the pharmacological Rev-Erbα activator SR9009, wild type (WT) mice showed reduced levels of pro-inflammatory transcripts. Additionally, in a glial cell model containing microglia and astrocytes from postnatal WT mice, Rev-Erbα knockdown increased neuronal death when OS was induced with hydrogen peroxide (Griffin et al., 2019). However, results in the 5xFAD mouse model of β-amyloidosis show a conflicting role for REV-ERBs in microglial activation. Here, a dramatic decrease in Aβ plaque burden is shown in REV-ERBα deficient mice, with no impact on APP expression when compared to WT. Additionally, inhibition of REV-ERBα *in vitro* not only showed an overall increase in Aβ uptake, but that uptake is increased in a CT-dependent manner which correlates with upregulated BMAL1 expression (Lee et al., 2020). Together, these findings suggest that Rev-Erbα plays a pivotal neuroprotective role against OS and an excessive neuroinflammatory response, although this relationship is confounded by conflicting results concerning REV-ERB’s regulation of microglial activation.

**FIGURE 2:**
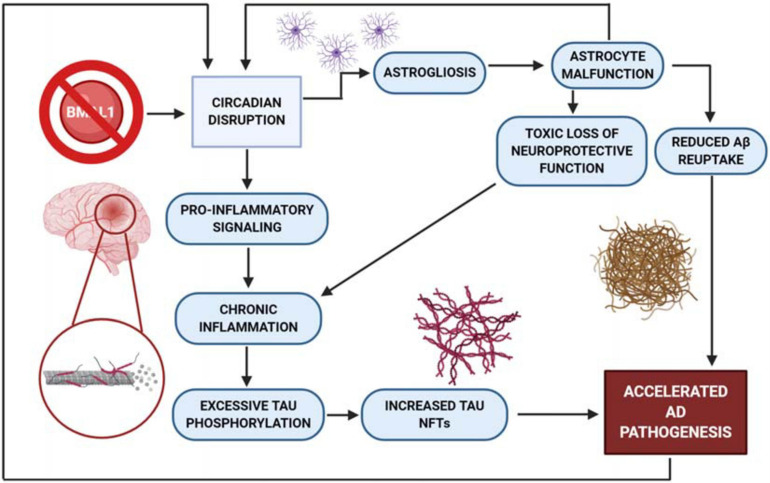
BMAL1 knockout mouse model produces increased astrogliosis. Knock out of BMAL1 drives excess astrogliosis, contributing to the acceleration of AD pathogenesis by way of astrocyte malfunction. This malfunction produces a loss of neuroprotection and reduces the astrocyte’s ability to transport Aβ out of the cell. Without protection from astrocytes, neuroinflammation then drives excessive pro-inflammatory signaling and tau phosphorylation, while producing an increased quantity of NFT’s. AD pathogenesis has also been shown to increase circadian dysfunction which further perpetuates neuronal damage and death.

There is a toxic cycle of protein accumulation and pro-inflammatory signaling that is exacerbated by clock dysfunction in neurodegenerative diseases. For example, tau NFTs are one source of the persistent pro-inflammatory response in neurodegeneration, as tau misfolding produces axon degeneration and eventual neuron death. It is postulated that protein unfolding events in several neurodegenerative disorders can also be triggered by microglial activation (Guzman-Martinez et al., 2019; Maccioni et al., 2020). This pro-inflammatory response serves as positive feedback for overstimulation of glial cells, which are thought to be involved in the excessive phosphorylation of tau (Guzman-Martinez et al., 2019). This increased neuroinflammatory stress accelerates tau pathology, and also gives rise to an alternative AD-specific conformation of astrocytes (Habib et al., 2020). Typically, astrocytes offer a neuroprotective function against excessive inflammation. However, altered functionality may have an effect on the function of the astrocytes, including toxic gains of function, or loss of function in neuronal support and Aβ plaque uptake and clearance (Garwood et al., 2017; Gonzalez-Reyes et al., 2017). Similar cycles have been demonstrated in all other proteinopathies discussed – toxic a-synuclein, TDP-43, and huntingtin proteins all drive neuroinflammation and are, in turn, exacerbated by the inflammatory response (Figure 2) (Fellner et al., 2011; Shi et al., 2010; Correia et al., 2015; Crotti and Glass, 2015; Lopes da Fonseca et al., 2015; Pearce et al., 2015; Jung and Chung, 2018; Spiller et al., 2018; Tremblay et al., 2019). Independent of which proteinopathy is being examined, the protein accumulation-driven neuroinflammatory response stimulates microglia to release cytokines that further drive a pro-inflammatory response, resulting in chronic inflammation and eventual neuronal death (Guzman-Martinez et al., 2019). It is also important to note the proposed circadian rhythmicity observed in glymphatic influx and clearance in mice, where drainage and influx of CSF from the cisterna magna appear to be time-of-day dependent (Hablitz et al., 2020). As previously demonstrated, the molecular clock exerts tight control over immune activity (Segal et al., 2018). The activation and inhibition mechanisms described by Segal et al. (2018), combined with the interactions of neuroinflammation, toxic protein aggregation and neuronal damage, suggest an overarching relationship between proteinopathy, neuroinflammatory stress, and the molecular clock. It is also important to remember that the clock is impacted by stress, as discussed earlier in this review. In a self-perpetuating cascade, circadian dysfunction both accelerates tau pathogenesis and is negatively impacted by tau toxicity and the chronic stress response. This feedback could explain the steep acceleration of cognitive decline often seen in laboratory models of proteinopathy-associated dementias (Baron et al., 2014).

## Discussion

**FIGURE 3:**
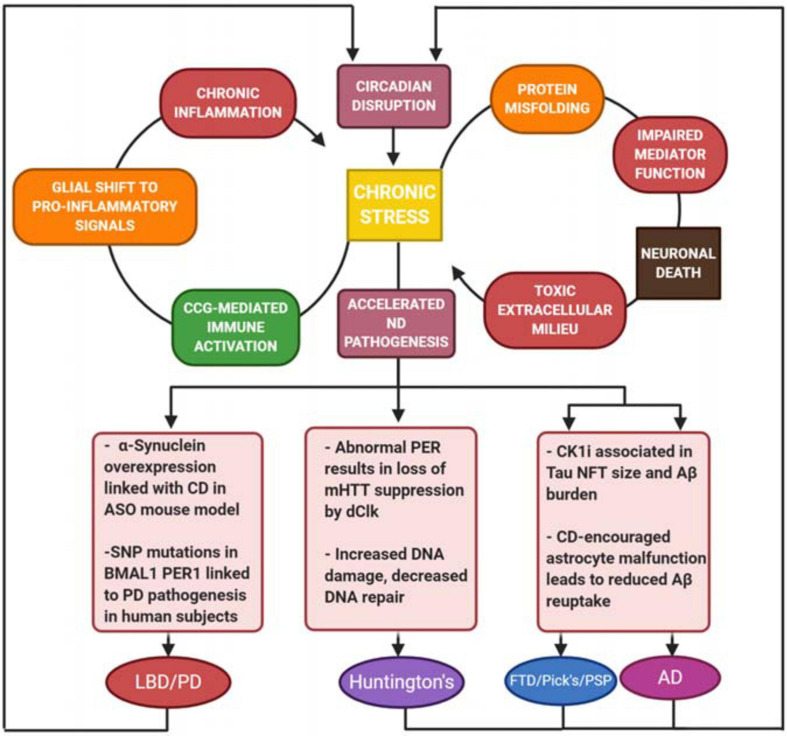
Harmful positive feedback as a driver for neurodegeneration. Stress and toxic extracellular milieu produced by neuronal death drives a chronic inflammatory response, as well as further breakdown of circadian function in cells. There is a common link between neurodegenerative disease (ND) and overactivity of the immune system, which plays a key role in each of the ND’s described. These conditions result in not only increased cell death, but deregulation of protein folding, increased DNA damage, astrocyte malfunction, and increased Aβ burden. There is a common link between ND and overactivity of the immune system, which plays a key role in each of the ND’s described. Additionally, symptoms of circadian dysfunction are often reported in ND patients, suggesting that the pathology of the disease also drives further CD, creating a devastating circular relationship between CD and neuronal damage.

This review summarizes findings from studies examining the effects of neurodegeneration and its associated pathology on the core clock (Table 1). Numerous findings indicate stress and neuroinflammation as a central process that links both circadian dysfunction and neurodegenerative disease (Figure 3). Perhaps the most compelling findings are the association of CK1δ with pathogenic tau deposits and the roles of Rev-Erbα in the neuroinflammatory response. Rev-Erbα presents itself as an interesting pharmacological treatment target to rescue neuronal damage, which would be an incredible step toward slowing neurodegenerative disease pathogenesis. The Rev-Erbα activator SR90009 has exhibited promising results in decreasing Aβ levels in the brain, improving synaptic health and axonal structure, as well as reversing cognitive defects in an AD mouse model (Roby et al., 2019). Additionally, our work demonstrating rescue of pathology, memory deficits, and circadian dysfunction in a mouse model of beta-amyloidosis by inhibiting CK1 activity suggests that targeting post-translational mechanisms is also a viable strategy – especially since kinases, such as CK1, that work on both pathogenic and clock mechanisms act as a nexus between the two processes, which potentially addresses the neurodegenerative disease pathogenesis and circadian dysfunction simultaneously.

**TABLE 1:** ND’s and ND Risk factors have been associated with the molecular clock in numerous studies.

Studied ND/ND Risk factor	Clock Gene(s) examined	Model system(s)	Observations	Investigator	DOI
AD	BMAL1, CBP, PER2	5XFAD mouse; HT22 cells	• BMAL1 and PER2 mRNA levels significantly altered abnormal oscillations in SCN of 5XFAD mice • No significant changes in CBP mRNA levels • Aβ induces BMAL1 and CBP degradation via SUMOylation *in vitro* • Disruptions the oscillation of PER2 mRNA levels • SUMO1 knockdown rescued BMAL1 degredation in cells	Song et al., 2015	10.1186/s13024-015-0007-
	BMAL1, CLOCK, PER1, PER2	Mesocricetus auratus	• BMAL1 and CLOCK expression attenuated with age in the SCN • PER1 and PER2 are not affected when housed in DD • PER1 less susceptible to photic induction with age in DD	Kolker et al., 2003	10.1177/074873040325180
	BMAL1	Primary human fibroblasts; postmortem human AD brain samples; NIH3T3 cells	• Rhythmic methylation of BMAL1 is altered in both AD brains and fibroblasts • E4 treatment with either 5-Aza-2′ deoxycytidine (DNA methyltransferase inhibitor) or *S*-adenosyl-methionine (methyl donor); NIH3T3 cells only ↑ methylation from methyl donor led to ↓ amplitude, longer periods, and phase delays in circadian rhythmicity; ↓ methylation from inhibitor led to shorter period length, advanced phase and ↑ amplitude	Cronin et al., 2017	10.1016/j.jalz.2016.10.00
	BMAL1, PER2	Mesocricetus auratus, Tg4510 mouse	• Aging attenuates SCN BMAL1 expression but does not affect musculoskeletal BMAL1 levels • Per2 mRNA had ↓ diurnal rhythms • Altered hippocampal BMAL1 expression • Altered hippocampal and hypothalamic Per2 expression	Duncan et al., 2013; Stevanovic et al., 2017	10.1016/j.brainres.2012.11.00, 10.1016/j.expneurol.2017.04.01
	Ck1δ, Ck1ε, PER1	APP-PS1 mouse	• PER1 expression abnormally high in vehicle treated transgenic mice compared to controls • Selective CK1i inhibitor PF-670462 produced dose dependent reduction of amyloid beta burden in both transgenic and non-transgenic mice • PF-670462 ↓ tau plaque size • PF-670462 rescued PER1 expression in transgenic mice • PF-670462 improved period length and rescued some cognitive function	Sundaram et al., 2019	10.1038/s41598-019-50197-
	CK1δ/ε	Human subjects (post mortem brain samples)	• CK1δ expression had 33-fold increase and CK1ε expression had a 9-fold increase in the CA1 region of the hippocampus • Both CK1 isoforms colocalize in NFTs and neuritic plaques	Ghoshal et al., 1999	10.1016/S0002-9440(10)65219-
	Dblt, dClk	*Drosophila melanogaster*	• Spag knockdown lowers Dblt and increases period length • Loss of function mutation in dClk; activation of Dronc • Dronc mediated tau cleavage and ↑ neurodegeneration	Means et al., 2015	10.1371/journal.pgen.100517
	PER1, PER2	C57BL/6 mouse	• Aβ 31-35 alters PER1 and PER2 expression in both core clock tissue (SCN) and peripheral cardiovascular tissues	Wang et al., 2016	10.1016/j.brainres.2016.03.02
	PER	*Drosophila melanogaster*	• PER expression is perturbed by ↑ β-cleavage of endogenous APPL • Overexpression of APPL maintained robust circadian rhythms in aged flies • PER KO flies had greater accumulation of oxidative damage • Impaired antioxidative stress responses also ↑ circadian dysfunction, neurodegeneration, and shortened lifespans	Blake et al., 2015; Krishnan et al., 2009	10.1016/j.nbd.2015.02.01, 10.18632/aging.10010
	Rev-Erba	SAMP8 mice	• Treatment with SR9009 injections ↓ AB levels in brains of treated mice, reversal of cognitive deficits, improved synaptic health and axoskeletal structure.	Roby et al., 2019	10.1371/journal.pone.021500
AD, aging	BMAL1, Ck1ε, CLOCK CRY1-2, PER1-3, RORα	3 × Tg-AD mouse	• All studied clock genes were affected by either aging or genotype • Effects are highly differentiated	Bellanti et al., 2017	10.3233/JAD-16094
	eval, pdf, PER, TIM	*Drosophila melanogaster*	• Pan-neuronal expression of Aβ causes progressive loss of circadian behavioral rhythmicity • Entrainment of the central molecular clock by exposure to regular light-dark cycles, even in the face of behavioral arrhythmia, prolongs the flies’ lifespan	Chen et al., 2014	10.1242/dmm.01413
	PER	*Drosophila melanogaster*	• KO of PER does not affect Aβ mediated pathologies (neurodegeneration, motor dysfunction) • Loss of rest/activity rhythms occurred while PER oscillations remained normal in Aβ expressing flies	Long et al., 2014	10.1371/journal.pone.010606
AD, PD, PSP, PiD	CK1δ	Human subjects	• CK1δ associates with Pick bodies in PiD and Tau containing neurofibrilary tangles in the remaining two tauopathies	Schwab et al., 2000	10.1016/s0197-4580(00)00110-
Aging, neuroinflammation, oxidative stress	BMAL1, CLOCK, NPAS2, PER1, PER2	BMAL1±; NestinCre+; BMAL1f/f; CLOCK KO; NPAS2/CLOCK DKO; PER1m/PER2m mice	• Ablation of BMAL1, CLOCK, NPAS2 caused severe reactive astrogliosis • Ablation of BMAL1 led to degeneration of synaptic terminals, neuronal oxidative damage and impaired expression of several redox defense genes	Musiek et al., 2013	10.1172/JCI7031
	BMAL1, CLOCK, CRY1, CRY2, PER1, PER2	BMAL1^–/–^ mouse; CRY1,2^–/–^ mouse; CLOCK/CLOCK mouse	• BMAL1 KO impairs PER and CRY expression, increases ROS production and leads to chronic oxidative stress in the brain • BMAL1, CLOCK, CRY1, or CRY2 deficiency may alter habituation, exploratory activity, or open field behaviors	Kondratova et al., 2010	10.18632/aging.10014
	BMAL1	BMAL1^–/–^ mouse; L929 cells	• Aged BMAL1 KO mice had age-dependent sarcopenia and bone loss, and ↑ ROS accumulation in various peripheral tissues which correlated with age-dependent degeneration • Cells with suppressed BMAL1 expression had ↓ PER1 expression	Kondratova et al., 2010	10.1101/gad.143220
	dClck, PER, TIM	*Drosophila melanogaster*	• PER protein expression ↓ with age • Expression of stress response genes is dependent on dClk	Kuintzle et al., 2017	10.1038/ncomms1452
Circadian Disruption	BMAL1, BMAL2	BMAL2 transgenic mice (B2Tg)	• Constitutive promoter expression of BMAL2 restores rhythmic locomotor activity and rhythmic metabolic processes in BMAL1 ablated mice	Shi et al., 2010	10.1016/j.cub.2009.12.03
	Dblt	*Drosophila melanogaster*	• Mutation of Dblt near phosphate recognition site or nuclear localization site shortens period length	Venkatesan et al., 2019	10.3390/ijms2004081
	PER	*Drosophila melanogaster*	• Mutation in PER causes impaired short-term and 24 h memory performance, shortened sleep cycles, and long-term memory deficits	Fropf et al., 2018	10.1016/j.nlm.2018.02.01
HD	dClk, PER, TIM	*Drosophila melanogaster*	• Partial knockdown of dClk suppressed mutant Huntingtin protein aggregation, this effect lost in absence of PER • PER expression ↓ during its normal peak hours • PER and TIM had prolonged expression patterns	Xu et al., 2019; Farago et al., 2019	10.1016/j.celrep.2019.03.01, 10.1038/s41598-019-43612-
	BMAL1, PER1-2	R6/2 mouse	• R6/2 mice have abnormally rapid clearance of PER1 and PER2 proteins *in vivo* • SCN brain slices have normal circadian gene expression • Alprazolam treatment resulted in ↑ PER2 mRNA levels and some improvements in motor function and survivability • Hepatic BMAL1 and PER2 expression maintained rhythmicity, though PER2 expression was significantly phase advanced • Hepatic CRY1 rhythmicity ablated • Listed clock genes ↑ compared to the untreated R6/2 mutants with restricted feeding • PER2 mRNA oscillations prematurely truncated during normal peak times • These alterations accompanied disturbed circadian behavior and eventual total circadian disintegration	Pallier et al., 2007; Maywood et al., 2010; Morton et al., 2005	10.1523/JNEUROSCI.0649-07.200, 10.1523/JNEUROSCI.1694-10.201, 10.1523/JNEUROSCI.3842-04.200
PD	BMAL1, CLOCK, CRY1, PER2, RORα	6-OHDA treated rats (PD phenotype); 6-OHDA treated SH-SY5Y cells	• BMAL1, PER2, and CLOCK mRNA levels ↓ and RORα mRNA levels ↑ in 6-OHDA rats • 6-OHDA-treated cells showed ↓ mRNA levels of BMAL1, CLOCK, PER2, and RORα, lower BMAL1/Clock protein expression, and ↑ BMAL/Clock binding ratio • BMAL1 acetylation ↑ in rats and cells treated with6-OHDA • Acetylated BMAL1 levels, CRY1 and PER2 mRNA levels partially rescued by Resveratrol in cells	Wang et al., 2018	10.1155/2018/485473
	PER2	ASO mouse	• SCN PER2 expression normal • SCN neurons had lower daytime firing rates • Circadian locomotor activities degenerated with age • Light adaptation response unaffected	Kudo et al., 2011	10.1016/j.expneurol.2011.08.00
	BMAL1, PER2, Rev-Erbα	Human subjects	• Peripheral BMAL1 expression had ↓ time-dependent variation • Brief increase in PER2 and Rev-Erbα at 4 AM, but otherwise no significant variation	Breen et al., 2014	10.1001/jamaneurol.2014.6
	BMAL1, CLOCK, CRY1-2, NPAS2, NR1D1, RORB	Human subjects	• BMAL1 and PER1 significantly associated with PD risk • SNPs in BMAL1 and PER1 also associated with PD	Gu et al., 2015	10.1038/srep1589
	BMAL1-2, CLOCK, DEC1	Leukocytes from human subjects	• BMAL1 and BMAL2 ↓ in PD patients	Ding et al., 2011	10.1016/j.neulet.2011.05.08
	BMAL1, CLOCK, CRY1-2 NPAS2, PER1-2	Leukocytes from human subjects	NPAS2 promoter significantly hypomethylated in PD patients compared to healthy controls • Both groups had some CRY1, insignificant BMAL1 methylation • No detectable methylation in PER1, PER2, CRY2, and CLOCK promoters	Lin et al., 2012	10.1016/j.neulet.2011.12.00
	BMAL1, CLOCK, CRY1-2, PER1-3,	Fibroblasts from two human subjects (P1 and P2)	• P1 fibroblasts had upregulated CLOCK, CRY1, and CRY2, expression and downregulated PER2 expression • P2 fibroblasts downregulate PER3 and CRY2 • CRY1, PER2, and PER3, had significant changes in oscillatory amplitude for both patients	Pacelli et al., 2019	10.3390/ijms2011277
PD, aging	BMAL1, CLOCK, CRY1, PER2, Rev-Erba, Rorα	C57BL/6 mouse; BSKO mouse; Sir2d mouse; BSTG mouse; N2a cells	• BMAL1, PER2, and Sirt1 ↓ in SCN of aged C57 mice which caused light entrainment impairment, disrupted circadian behaviors, and longer periods • BMAL1 and PER2 mRNA levels ↓ in Sirt1 KO mice (BSKO), behavioral disturbances observed in aged C57s, recapitulated in BSKOs • BSTG mice had ↑ BMAL1 and PER2 levels • All listed clock genes regulated by SIRT1 in N2a cells • KO of SIRT1 *in vitro* ↓ mRNA levels of all listed clock genes	Chang and Guarente, 2013	10.1016/j.cell.2013.05.0
PD, neuroinflammation	BMAL1, CLOCK, CRY1, CRY2, DBP, PER1, PER2, Rev-ERB, Rorα	Sprague-Dawley rats treated with LPS (lipopolysaccharide), ROT (rotenone), or both to induce PD phenotype	• LPS injected rats sustained dopaminergic neuron loss and severe neuroinflammation • BMAL1 protein and mRNA expression ↓ in all groups • CLOCK and NPAS2 mRNA levels ↓ in all groups • PER1, PER2 ↓ in all groups • CRY1 and CRY2 expressions not affected in any group • REV-ERB α and RORα↓ in protein expression and mRNA levels in all groups	Li et al., 2019	10.1007/s12640-018-9968-
DLB	BMAL1, CLOCK, CRY1, CRY2, PER1, PER2, PER3, TIM, CK1ε	Leukocytes from human subjects	• PER1 and CRY1 circadian genes methylated • DLB patients had significantly ↑ gene methylation	Liu et al., 2008	10.1016/j.neulet.2008.02.04

*Here several studies and their observations are summarized to portray the multiple connections found between the clock and ND, and to outline the significant impact understanding this relationship may have in ND treatment.*

Although significant progress has been made over the past decade, there are many lingering questions about the complex relationship between the molecular clock and the pathogenic mechanisms of neurodegeneration. Answers to these questions may lead to improved diagnosis, treatment, and management of these debilitating diseases.

## Author Contributions

HJ and BC wrote the early drafts and designed the figures. DG and JG were responsible for writing and editing the text and figures. All authors contributed to the article and approved the submitted version.

## Conflict of Interest

The authors declare that the research was conducted in the absence of any commercial or financial relationships that could be construed as a potential conflict of interest.
